# B cells from rheumatoid arthritis patients show important alterations in the expression of CD86 and FcγRIIb, which are modulated by anti-tumor necrosis factor therapy

**DOI:** 10.1186/ar2985

**Published:** 2010-04-15

**Authors:** Diego Catalán, Octavio Aravena, Francisca Sabugo, Pamela Wurmann, Lilian Soto, Alexis M Kalergis, Miguel Cuchacovich, Juan C Aguillón

**Affiliations:** 1 Programa Disciplinario de Inmunología, Instituto de Ciencias Biomédicas (ICBM), Facultad de Medicina, Universidad de Chile, Avenida Independencia 1027, Santiago, Chile; 2 Sección de Reumatología, Departamento de Medicina, Hospital Clínico, Universidad de Chile, Santos Dumont 999, Santiago, Chile; 3 Departamento de Genética Molecular y Microbiología, Facultad de Ciencias Biológicas, Pontificia Universidad Católica de Chile, Av. Bernardo O'Higgins 340, Santiago, Chile

## Abstract

**Introduction:**

Several molecules help preserve peripheral B cell tolerance, but when altered, they may predispose to autoimmunity. This work studied the expression of the costimulatory molecule CD86 and the inhibitory receptor for IgG immune complexes FcγRIIb (CD32b), on B cells from rheumatoid arthritis (RA) patients, and the influence of anti-tumor necrosis factor (TNF) therapy.

**Methods:**

Peripheral B cells from 18 RA patients and 13 healthy donors were characterized using flow cytometry. Eleven patients who underwent a six-month adalimumab therapy were further assessed for phenotypic changes on their B cells.

**Results:**

RA patients exhibited a high percentage of naïve and memory B cells expressing CD86. In contrast, expression of FcγRIIb was significantly reduced on RA memory B cells and plasmablasts as compared to healthy donors, probably due to downregulation of this receptor when differentiating from naïve to memory cells. These alterations on FcγRIIb were associated with high levels of anti-citrullinated vimentin autoantibodies. In addition, treatment with adalimumab normalized the expression of CD86 on memory B cells and reduced the expression of FcγRIIb, mainly on naïve B cells.

**Conclusions:**

Our findings show that peripheral B cells from RA patients have an altered expression of key molecules, such as CD86 and FcγRIIb. Because this latter receptor is required for feedback inhibition, a deficient expression might contribute to humoral autoimmune responses. Furthermore, these molecules are likely to be influenced by inflammatory factors, since they were modulated by TNF inhibition.

## Introduction

Rheumatoid arthritis (RA) is a chronic, inflammatory, and autoimmune disease that affects mainly synovial joints, leading to progressive destruction, pain, and disability. It is well known from mouse models that B cells play a pivotal role in the development of the autoimmune process as a precursor of antibody-secreting cells but also as antigen-presenting cells (APCs) [1,2].

Immune cells express an array of receptors that bind the Fc portion of IgG-containing immune complexes (FcγRs). Particularly, it has been stated that B cells and plasma cells express only the low-affinity receptor FcγRIIb, which, in contrast to FcγRIIa, has an immunoreceptor tyrosine-based inhibitory motif on the cytoplasmic domain. This characteristic confers an inhibitory function to the receptor which is essential in several checkpoint stages in which abnormal humoral responses are quenched by mechanisms that include the deletion of autoreactive clones and feedback inhibition of IgG secretion [3].

Given this property, it is not surprising that these molecules have been involved in autoimmune processes. Autoimmune-susceptible mice present several polymorphisms in the regulatory regions of the FcγRIIb gene, which result in a reduced expression of the receptor on germinal center B cells [4]. Moreover, depending on the strain, mice deficient in FcγRIIb can spontaneously develop a lupus-like syndrome, become susceptible to collagen-induced arthritis (CIA), or develop a severe phenotype of CIA or experimental autoimmune encephalomyelitis [5-8]. In contrast, overexpression of FcγRIIb on B cells, but not on macrophages, leads to an early resolution of CIA and reduced spontaneous lupus [9].

On the other hand, human autoimmune diseases characterized by a deregulated secretion of autoantibodies, such as systemic lupus erythematosus (SLE) and RA, have been associated with abnormalities in FcγRIIb regulation. Polymorphisms in the promoter region as well as in the transmembrane domain of the FcγRIIb gene have been described to affect the expression and function of this receptor, respectively [10-12]. While both polymorphisms in *FcγRIIb *are associated with SLE occurrence [10,13], the one on the transmembrane domain is also associated with joint damage in RA [14]. Although alterations in the expression of FcγRIIb on B cells have been described for other autoimmune diseases [15-18], no data about RA are available.

The aim of our study was to evaluate the phenotype of B cells from RA patients, focusing on their activation status and their expression of FcγRIIb. These parameters were compared with those obtained from B cells of healthy individuals. In addition, we followed up on these patients during anti-tumor necrosis factor (anti-TNF) therapy and assessed the phenotype of their B cells after 6 months of treatment. Our findings show that B cells from RA patients are activated, as reflected by the expression of CD86. We have also observed an altered expression of FcγRIIb, which is associated with the presence of autoantibodies. These abnormalities were shown to be partially reverted by anti-TNF therapy.

## Materials and methods

### Patients

We recruited 18 patients meeting the American College of Rheumatology criteria for RA [19]. All of the patients were women, with a mean ± standard deviation (SD) age of 52.8 ± 10.5 years and disease duration of 16.3 ± 7 years at study entry. All of them exhibited an active disease defined as at least six swollen joints, at least nine tender joints, and morning stiffness for more than 1 hour, regardless of being under treatment with disease-modifying antirheumatic drugs. Disease activity was determined based on the disease activity score for 28 joints (DAS-28) [20]. Thirteen patients received 40 mg of adalimumab (kindly provided by Abbott Laboratories, Abbott Park, IL, USA) subcutaneously every other week during 24 weeks. The European League Against Rheumatism (EULAR) response criteria were used to establish the degree of response to treatment [21]. Thirteen healthy women were recruited as a control group, with a mean ± SD age of 39.4 ± 9.7 years. For the analyses of CD86 expression, we had available samples of only 8 of the 13 healthy donors. Blood samples for flow cytometry analyses and serum determinations were drawn from RA patients at study entry and 6 months after beginning adalimumab administration. The study was approved by the Ethical Committee of the Hospital Clínico Universidad de Chile, and all patients and controls gave their written informed consent.

### Serum antibody determination

Serum antibodies against modified and citrullinated vimentin (anti-MCV) were measured using a commercial enzyme-linked immunosorbent assay (ELISA) kit (Orgentec, Mainz, Germany) in accordance with the instructions of the manufacturer. The cutoff level was set at 20 U/mL. Serum anti-cyclic citrullinated peptide (ccp) antibodies were detected using a commercial second-generation ELISA (Axis-Shield, Dundee, Scotland) in accordance with the instructions of the manufacturer. A cutoff level of 5 U/mL was considered. Total serum IgG levels were measured by ELISA at the Immunology Laboratory of the Hospital Clínico Universidad de Chile, and the range of concentrations considered normal was 639 to 1,349 mg/dL.

### B-lymphocyte phenotyping

**Figure 1:**
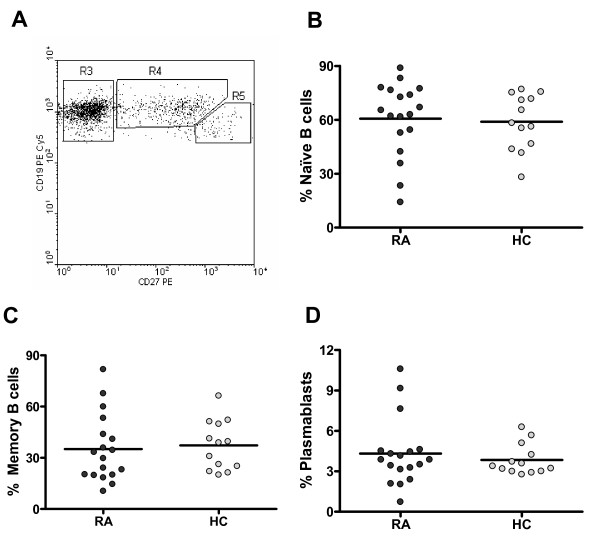
**Characterization of B-cell subpopulations from rheumatoid arthritis (RA) patients and healthy controls (HCs)**. **(a) **Dot plot representing the distribution of naïve B cells (R3), memory B cells (R4), and plasmablasts (R5) which was used on further analyses. No differences in the percentages of naïve B cells **(b)**, memory B cells **(c)**, and plasmablasts **(d) **between 18 RA patients and 13 HCs were detected. *P *> 0.05, two-tailed unpaired Student *t *test. Horizontal lines represent mean values.

We characterized B cells using the following monoclonal anti-human antibodies: anti-CD19 phycoerythrin (PE) cyanine 5 (Cy5), anti-CD27 fluorescein isothiocyanate (FITC), anti-CD27 PE, anti-CD86 FITC (BD Biosciences, San Jose, CA, USA), and anti-FcγRII PE (clone 7.3; Fitzgerald Industries International, Acton, MA, USA). For the staining procedure, anticoagulated whole blood was incubated with fluorescent antibodies for 30 minutes at 4°C. Subsequently, red cells were lysed with ammonium chloride potassium (ACK) buffer, washed, and fixed for flow cytometry (FACSCalibur; BD Biosciences, San Jose, CA, USA). Data were analyzed with WinMDI 2.9 Software (TSRI Flow Cytometry Core Facility, La Jolla, CA, USA). A region was set to define the lymphocytic population according to forward and side scatter patterns. B cells were defined as CD19-expressing cells, and a second region was set for them, while CD27 was used to discriminate memory from naïve subsets. Plasmablasts were defined as CD19^low ^CD27^high^-expressing cells (Figure 1a). Mean fluorescence intensity (MFI) was used as the analysis parameter for the expression of FcγRIIb.

### Statistical analyses

All of the study variables were tested for normality with the Shapiro-Wilk test. Differences between patient and control groups were analyzed using the two-tailed unpaired Student *t *test or Mann-Whitney *U *test, when appropriate. For comparisons between different B-cell subsets and for different adalimumab therapy time points, the two-tailed paired Student *t *test or Wilcoxon signed-rank test were used, when appropriate. Correlations were evaluated with a two-tailed Spearman correlation test. For contingency analyses, a two-sided Fisher exact test was used. *P *values of less than 0.05 were considered significant. For statistic analyses and graphics, GraphPad Prism 4 software (GraphPad Software, Inc., La Jolla, CA, USA) was used.

## Results

### Rheumatoid arthritis patients show peripheral B-cell frequencies similar to those of healthy controls

Abnormalities in the frequency of B cells and in the proportion of different B-cell subsets in autoimmune diseases have been previously reported [22,23]. Despite a broad dispersion, we found no significant differences between RA and control subjects when comparing the percentage of B cells in the lymphocytic population (mean percentage ± SD 7.5 ± 3.1 and 9 ± 2.2, respectively). Furthermore, when we discriminated B cells in naïve cells, memory cells and plasmablasts by means of CD19 and CD27 staining, we detected similar percentages of these subsets in the two groups of individuals (Figure 1).

### Higher frequency of activated naïve and memory B cells in rheumatoid arthritis patients than in healthy controls

**Figure 2:**
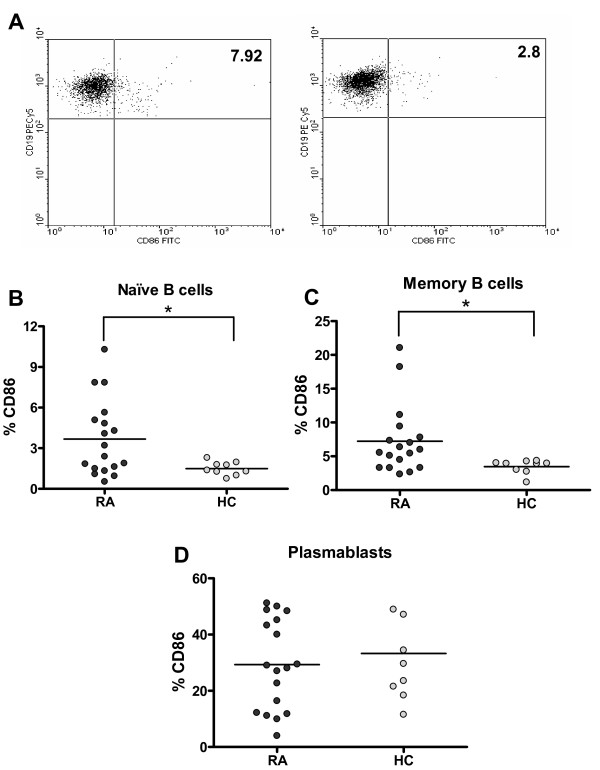
**Increased expression of CD86 on naïve and memory B cells from rheumatoid arthritis (RA) patients**. **(a) **Dot plots representative of the expression of CD86 on B cells from an RA patient (left) and a healthy control (HC) (right). The number in the quadrant represents the percentage of CD19^+ ^CD86^+ ^cells. Graphics summarizing the percentages of CD86^+ ^cells among naïve B cells **(b)**, memory B cells **(c)**, and plasmablasts **(d) **from 18 RA patients and 9 HCs. *P < 0.05, two-tailed Mann-Whitney *U *test. Horizontal lines represent mean values.

To assess the activation level of circulating B lymphocytes from RA patients, we analyzed CD86, a co-stimulatory molecule that increases its expression on B-cell surface upon activation [24,25]. We observed that RA patients have more CD86-expressing cells on the naïve and memory subsets than healthy controls do (*P *= 0.042 and *P *= 0.017, respectively), while no significant differences were observed for plasmablasts (Figure 2).

### Reduced FcγRIIb expression on memory B cells and plasmablasts from rheumatoid arthritis patients

**Figure 3:**
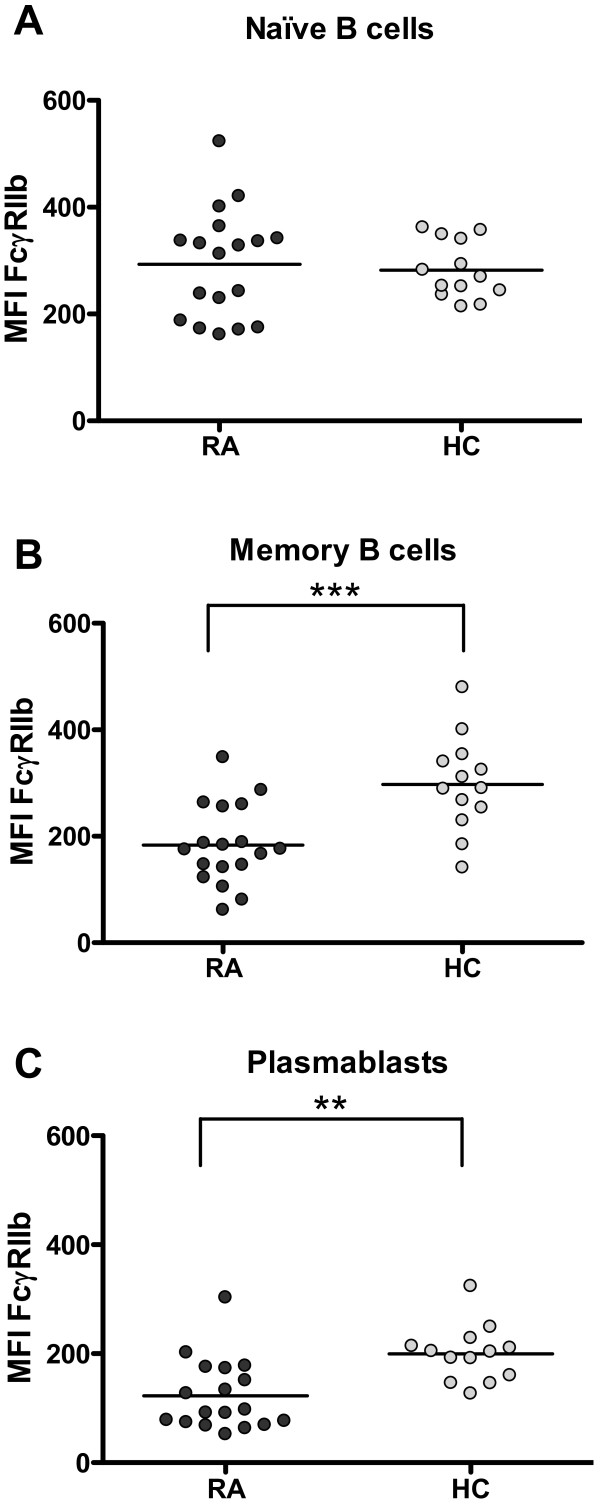
**Decreased expression of FcγRIIb on memory B cells and plasmablasts from rheumatoid arthritis (RA) patients**. Graphics summarize the expression of FcγRIIb on naïve B cells **(a)**, memory B cells **(b)**, and plasmablasts **(c) **from 18 RA patients and 13 healthy controls (HCs). Expression was quantified as mean fluorescence intensity (MFI). **P < 0.01, ***P < 0.001, two-tailed unpaired Student *t *test. Horizontal lines represent mean values.

As for CD86, the evaluation of FcγRIIb expression was carried out by analyzing each B-cell subset individually. We found that, although naïve B cells from RA patients and controls expressed similar levels of this receptor, its expression was significantly lower on RA memory B cells and plasmablasts (*P *= 0.0005 and *P *= 0.0013, respectively) (Figure 3). No correlations were observed between FcγRIIb expression on B cells and disease activity or percentage of CD86-expressing B cells (data not shown).

**Figure 4:**
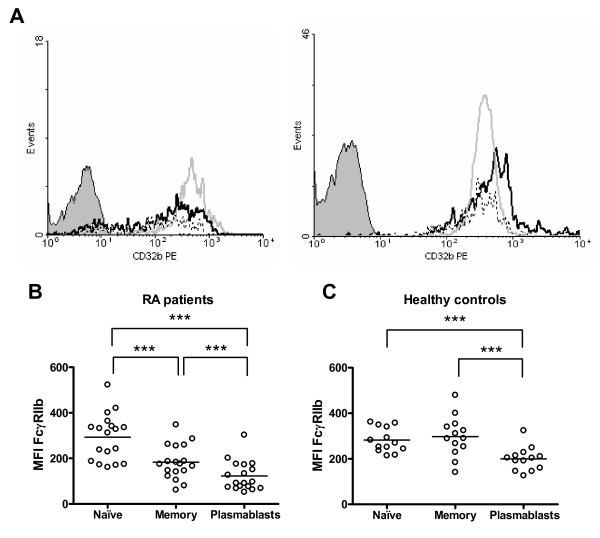
**Altered regulation of FcγRIIb on B cells from rheumatoid arthritis (RA) patients**. **(a) **Representative histograms of FcγRIIb (CD32b) expression on naïve B cells (gray line), memory B cells (black line), and plasmablasts (dotted line) from an RA patient (left) and a healthy control (right). The shaded curve represents the isotype control. Graphics show a comparison of FcγRIIb expression between naïve B cells, memory B cells, and plasmablasts from 18 RA patients **(b) **and 13 healthy controls **(c)**. Expression was quantified as mean fluorescence intensity (MFI). The differences in the FcγRIIb expression levels between B-cell subpopulations were analyzed with the two-tailed paired Student *t *test; ***P < 0.001. Horizontal lines represent mean values. PE, phycoerythrin.

There is evidence of a normal upregulation of FcγRIIb following naïve B-cell activation and differentiation to a memory cell [15]. In our sample of healthy subjects, we detected that most individuals upregulate the expression of FcγRIIb from naïve to memory B cells (8/13), in contrast with the RA group, in which 15 out of 18 patients were downregulators (ΔMFI of greater than 10 between naïve and memory populations) (*P *= 0.029). This decrease of FcγRIIb expression on memory B cells as compared with naïve B cells from RA patients was statistically significant (*P *= 0.0001) (Figure 4b). We also noticed that RA patients show a further decrease in FcγRIIb expression on plasmablasts in comparison with memory B cells (*P *= 0.0001) (Figure 4b). A similar reduction was observed in healthy controls (*P *= 0.0002) (Figure 4c), although the expression levels reached by the healthy controls were still higher than those exhibited by RA patients (Figure 3c).

### FcγRIIb expression on B cells is associated with autoantibody levels

**Figure 5:**
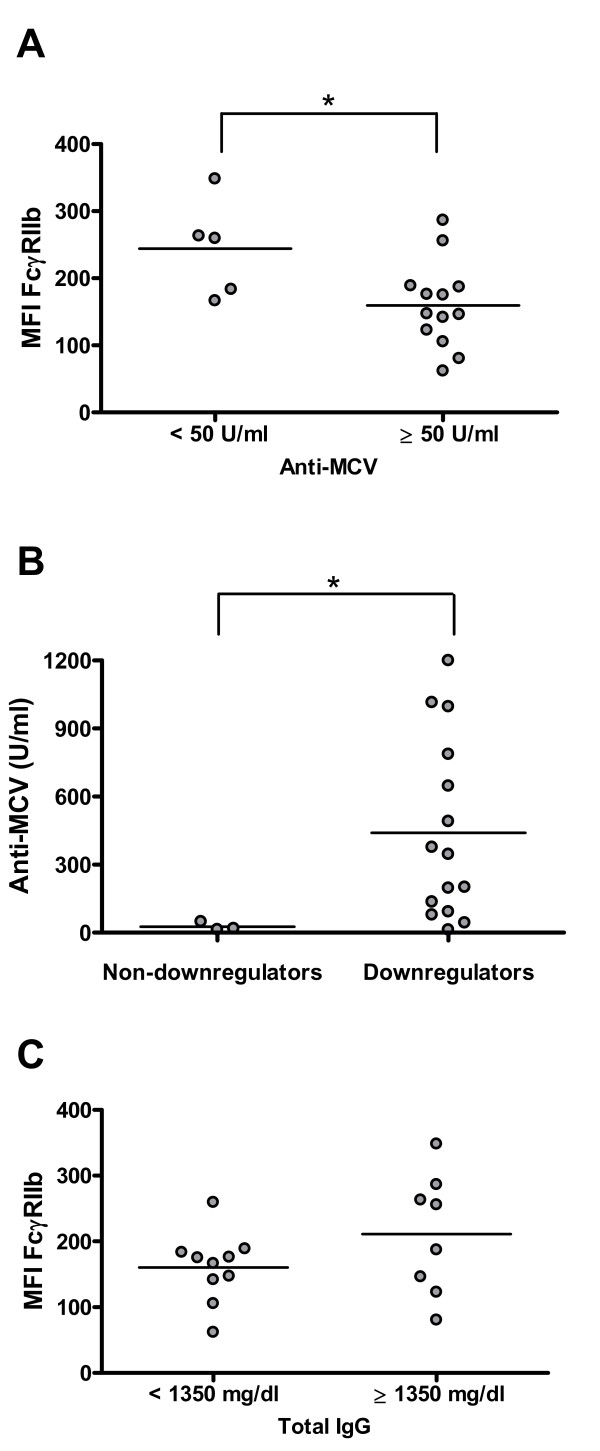
**FcγRIIb expression levels on rheumatoid arthritis (RA) patients' memory B cells are inversely associated with anti-modified and citrullinated vimentin (anti-MCV) titers**. **(a) **FcγRIIb expression on memory B cells from RA patients with high titers (at least 50 U/mL) and from those with no or low titers (less than 50 U/mL) of serum anti-MCV antibodies. Expression was quantified as mean fluorescence intensity (MFI). *P < 0.05, two-tailed Mann-Whitney *U *test. **(b) **Anti-MCV antibody titers in patients who downregulated the expression of FcγRIIb (the difference between MFI of naïve B cells and MFI of memory B cells was greater than 10 for downregulators) and in those who upregulated or maintained it almost invariable (the difference between MFI of naïve B cells and MFI of memory B cells was not greater than 10 for non-downregulators). *P < 0.05, two-tailed Mann-Whitney *U *test. **(c) **FcγRIIb expression on memory B cells from RA patients with normal levels (less than 1,350 mg/dL) and high levels (at least 1,350 mg/dL) of total serum IgG. Expression was quantified as MFI. *P *> 0.05, two-tailed Mann-Whitney *U *test. Horizontal lines represent mean values.

As one of the main functions of FcγRIIb on B cells is to control the development of autoimmunity by providing feedback inhibition in order to limit the secretion of autoantibodies, we assessed whether the levels of autoantibodies on RA patients were related to the expression of this inhibitory receptor on B cells. For this purpose, we measured serum anti-MCV antibodies since they have been described to be highly specific for RA [26]. Interestingly, RA patients negative for serum anti-MCV antibodies or with low levels (less than 50 U/mL) displayed a higher expression of FcγRIIb but only on memory B cells (*P *= 0.048) (Figure 5a). Also, we found that all three patients who did not downregulate FcγRIIb from naïve to memory B cells exhibited no or very low titers of anti-MCV antibodies (*P *= 0.033) (Figure 5b). We obtained similar results when we measured anti-ccp antibodies (data not shown). To evaluate whether this association was restricted to autoantibodies, we compared FcγRIIb expression on memory B cells in patients with normal (less than 1,350 mg/dL) or high (at least 1,350 mg/dL) levels of total serum IgG without detecting significant differences between the two groups (Figure 5c).

### Anti-tumor necrosis factor therapy can influence B-cell phenotype in rheumatoid arthritis patients

**Figure 6:**
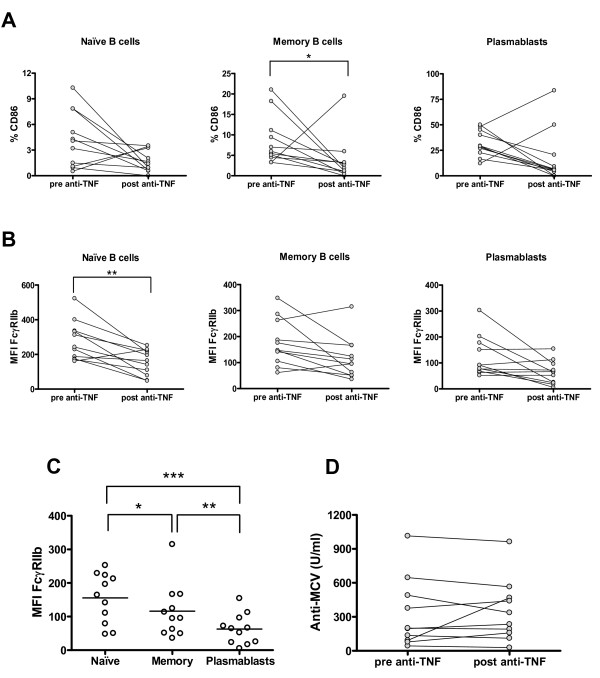
**B-cell phenotype and anti-modified and citrullinated vimentin (anti-MCV) titers on rheumatoid arthritis (RA) patients after 6 months of adalimumab therapy**. After 6 months of anti-tumor necrosis factor (anti-TNF) therapy, B-cell phenotype and serum anti-MCV antibodies from 11 RA patients, who exhibited at least a moderate response to the treatment, were reassessed. **(a) **Graphics summarizing the percentages of CD86^+ ^cells among naïve B cells, memory B cells, and plasmablasts from RA patients before and after 6 months of anti-TNF therapy. *P < 0.05, two-tailed Wilcoxon signed-rank test. **(b) **Graphics summarizing FcγRIIb expression on naïve B cells, memory B cells, and plasmablasts from RA patients before and after 6 months of anti-TNF therapy. Expression was quantified as mean fluorescence intensity (MFI). **P < 0.01, two-tailed paired Student *t *test. **(c) **Comparison of FcγRIIb expression between naïve B cells, memory B cells, and plasmablasts from RA patients after anti-TNF therapy. The differences in FcγRIIb expression levels between B-cell subpopulations were analyzed with the two-tailed paired Student *t *test; *P < 0.05, **P < 0.01, ***P < 0.001. **(d) **Comparison of serum anti-MCV antibody levels before and after 6 months of anti-TNF therapy. *P *> 0.05, two-tailed paired Student *t *test. Horizontal lines represent mean values.

Next, we wanted to evaluate whether the alterations observed on RA patients' B cells could be reverted by the treatment with adalimumab. Of the 13 RA patients who completed 6 months of treatment with an anti-TNF antibody (adalimumab), only 11 exhibited at least a moderate response according to the EULAR response criteria. In these patients, the percentage of total B cells as well as the proportion of naïve, memory, or plasmablast subsets remained unchanged (data not shown). However, the anti-TNF therapy caused a decrease in the proportion of memory B cells expressing CD86 after 6 months of therapy (*P *= 0.032) (Figure 6a). Notably, the change affecting CD86 paralleled a reduction in the intensity of FcγRIIb expression, but this decrease reached significance on the naïve B-cell subpopulation only (*P *= 0.003) (Figure 6b). Consequently, there was an attenuation of the receptor downregulation observed before adalimumab treatment was started (Figure 4b), but the difference between naïve and memory B cells was still significant (*P *= 0.046) (Figure 6c). In addition, anti-MCV antibody titers remained stable throughout this period (Figure 6d).

## Discussion

In the present work, we provide evidence that phenotypic alterations on B cells from RA patients affect key molecules involved in the regulation of antigen presentation and antibody secretion functions. A major role of B cells in the development and perpetuation of RA has been consistently demonstrated with the appearance of B cell-depleting therapy and its impressive results in reducing symptoms and preventing disease progression [27]. Studies on murine models have suggested that antigen-specific B cells are required as APCs for the induction of autoimmune arthritis, owing to their expression of MHC (major histocompatibility complex) class II and co-stimulatory molecules CD80 and CD86 [28,29]. CD86 is upregulated on activated B cells upon B-cell receptor (BCR) and CD19/CD21 complex engagement [24,25]. Our results show that RA patients have a higher proportion of naïve and memory B cells expressing CD86 than healthy controls, reflecting an expanded activated status within these subpopulations, which is likely to favor a more productive interaction with pathogenic T cells. Analogous results have been reported for other inflammatory diseases, such as SLE [30-33], systemic sclerosis [34], asthma [35], and irritable bowel syndrome [36]. Presumably, constant stimulation of autoantigens through BCR and the influence of other proinflammatory signals, such as stimulation through Toll-like receptors (TLRs) [37], give rise to this activated phenotype. Also, it is probable that the effect of these activation stimuli could be attenuated by the cross-linking of IgG-containing immune complexes to their inhibitor receptor, FcγRIIb, since studies on dendritic cells (DCs) have demonstrated that exposure to immune complexes together with a blockage of FcγRIIb is sufficient to induce an increase in the expression of CD86 [38,39], whereas the overexpression of this receptor on murine B cells reverses the induction of CD86 triggered via BCR [9]. However, in a multifactorial complex disease such as RA, it is expected that the expression of this or other activation markers is influenced by a variety of factors as it is suggested by the absence of correlation between CD86-expressing B cells and FcγRIIb expression levels found in our patients.

Our data show that RA patients present reduced levels of FcγRIIb on memory B cells and plasmablasts compared with healthy donors. This phenomenon can be explained by the abnormal downregulation of this receptor from naïve to memory B cells which we observed in our RA group. These results are concordant with those seen in SLE and chronic inflammatory demyelinating polyneuropathy, other autoimmune diseases characterized by uncontrolled secretion of autoantibodies [15-18].

It has been postulated that FcγRIIb upregulation might constitute a critical checkpoint in peripheral tolerance by providing an inhibitory feedback that limits the ongoing humoral response to self-antigens. In fact, in lupus-prone mice, the restoration of FcγRIIb levels on B cells can revert the secretion of autoantibodies and renal disease [40]. Furthermore, the expression of this inhibitory receptor specifically on B cells, but not on macrophages, can be determinant for controlling autoimmunity in models of arthritis and lupus [9]. Through this work, we provide new evidence that may help to reinforce this concept as we have revealed, for the first time, an association between high levels of FcγRIIb on memory B cells and no or low titers of specific autoantibodies, anti-MCV antibodies. This interesting result appears to be exclusive for autoimmune responses since we did not find a similar association when analyzing total IgG levels. It is noteworthy to consider that in this study we have examined only the expression of FcγRIIb, but not its function, which if altered could also affect the regulatory ability over the humoral response against citrullinated proteins. Likewise, a recent publication has demonstrated an association between a functional polymorphism for *FcγRIIb *and anti-ccp (+) RA in an Asian population [41].

After patients underwent 6 months of therapy with an anti-TNF antibody, we observed a decrease in CD86-expressing memory B cells, reflecting an attenuation of the B-cell activated status. Paradoxically, FcγRIIb expression on the naïve B-cell subset also decreased significantly. It has been demonstrated that FcγRIIa and FcγRIIb on human monocytes are differentially regulated by Th1/Th2 cytokines, with interferon-gamma favoring the activator receptor and interleukin-4 favoring the inhibitory receptor [42]. On the other hand, TNF downmodulates FcγRIIb and FcγRIIa on monocytes, not affecting FcγRIIa but reducing FcγRIIb expression on DCs, while increasing FcγRIIa without changing FcγRIIb on neutrophils, indicating a tight cell-specific regulation [43-47]. To our knowledge, no studies addressing the effect of TNF over FcγRIIb on human B cells have been published, but our results strongly suggest that TNF or other downstream cytokines may influence the expression of this receptor on B lymphocytes. In regard to RA, exposure of DCs to synovial fluid from RA patients has been shown to lead to an upregulation of FcγRIIb [14] and an elevated expression of FcγRIIb has been demonstrated on RA synovial tissue, probably counteracting the upregulation of other activating receptors [48]. Some studies have reported that FcγR expression levels on leukocytes can vary with anti-rheumatic drugs, which would promote a more inhibitory profile [48-52]. It is possible that the reduction in FcγRIIb expression that we observed on naïve B cells as a consequence of anti-TNF therapy is accompanied by a decrease in the expression of activating receptors on these cells, like TLR9 and CD21, which would determine a restoration of the protective activator/inhibitor balance, but this issue needs to be investigated. The effect of TNF blockage, however, was not sufficient to prevent the downregulation of FcγRIIb from naïve to memory B cells. These results are in accordance with the fact that our group of patients did not achieve a reduction in anti-MCV titers after 6 months of therapy. Others have reported that changes in these antibodies become significant after 18 months of anti-TNF therapy [53], so it is conceivable that a full normalization of B-cell phenotype may become apparent only over longer follow-up periods.

## Conclusions

Our data demonstrate the existence of important alterations in the phenotype of peripheral B cells from RA patients, involving the expression of the co-stimulatory molecule CD86 and the inhibitory receptor FcγRIIb, the latter being associated with high titers of autoantibodies. We consider that our study contributes relevant evidence to a better comprehension of the molecular mechanisms that are implied in the regulation of B cells and the role that they play in the autoimmune response elicited in RA.

## Abbreviations

APC: antigen-presenting cell; BCR: B-cell receptor; ccp: cyclic citrullinated peptide; CIA: collagen-induced arthritis; DC: dendritic cell; ELISA: enzyme-linked immunosorbent assay; EULAR: European League Against Rheumatism; FcγR: Receptor for the Fc region of IgG-containing immune complexes; FITC: fluorescein isothiocyanate; MCV: modified and citrullinated vimentin; MFI: mean fluorescence intensity; PE: phycoerythrin; RA: rheumatoid arthritis; SD: standard deviation; SLE: systemic lupus erythematosus; TLR: Toll-like receptor; TNF: tumor necrosis factor.

## Competing interests

The authors declare that they have no competing interests.

## Authors' contributions

DC participated in the design of the study, carried out the acquisition and analysis of data, and drafted the manuscript. OA participated in B-cell phenotyping. FS coordinated the recruitment of patients, performed the clinical evaluations, and helped with data analysis. PW participated in the recruitment and clinical evaluations of patients. LS participated in the recruitment of patients and clinical data analysis. MC participated in the design and coordination of the study. AMK participated in the conception and design of the study and critically revised the manuscript. JCA participated in the conception and design of the study and in the interpretation of data and helped to draft the manuscript. All authors read and approved the final manuscript.
